# Enhanced image sensing with avalanche multiplication in hybrid structure of crystalline selenium photoconversion layer and CMOSFETs

**DOI:** 10.1038/s41598-020-78837-7

**Published:** 2020-12-14

**Authors:** Shigeyuki Imura, Keitada Mineo, Yuki Honda, Toshiki Arai, Kazunori Miyakawa, Toshihisa Watabe, Misao Kubota, Keisuke Nishimoto, Mutsumi Sugiyama, Masakazu Nanba

**Affiliations:** 1 Science & Technology Research Laboratories, Japan Broadcasting Corporation (NHK), 1-10-11 Kinuta, Setagaya-ku, Tokyo, 157-8510 Japan; 2 Japan Broadcasting Corporation (NHK), Engineering System Inc., 1-10-11 Kinuta, Setagaya-ku, Tokyo, 157-8510 Japan; 3 Department of Electrical Engineering, Faculty of Science and Technology, Tokyo University of Science, 2641 Yamazaki, Noda, Chiba 278-8510 Japan

**Keywords:** Materials for devices, Materials science

## Abstract

The recent improvements of complementary metal–oxide–semiconductor (CMOS) image sensors are playing an essential role in emerging high-definition video cameras, which provide viewers with a stronger sensation of reality. However, the devices suffer from decreasing sensitivity due to the shrinkage of pixels. We herein address this problem by introducing a hybrid structure comprising crystalline-selenium (c-Se)-based photoconversion layers and 8 K resolution (7472 × 4320 pixels) CMOS field-effect transistors (FETs) to amplify signals using the avalanche multiplication of photogenerated carriers. Using low-defect-level NiO as an electric field buffer and an electron blocking layer, we confirmed signal amplification by a factor of approximately 1.4 while the dark current remained low at 2.6 nA/cm^2^ at a reverse bias voltage of 22.6 V. Furthermore, we successfully obtained a brighter image based on the amplified signals without any notable noise degradation.

## Introduction

Rapid advances in complementary metal–oxide–semiconductor (CMOS) image sensor technology have enabled the development of high-definition imaging systems for various applications, such as high-end broadcasting and consumer video cameras, advanced smartphone cameras, omnidirectional augmented/virtual reality cameras, and 3D reconstruction light field cameras. In particular, recent remarkable progress in mobile device cameras has accelerated the downsizing of pixels to the submicron scale and this trend is expected to continue. Although pixel shrinkage is advantageous for making compact and high-resolution cameras, the sensitivity of image sensors linearly decreases with the shrinkage of the pixel area due to the decreased amount of light received per pixel. Some structural approaches have been proposed to enhance sensitivity^1–3^; however, they will not solve the actual cause of the problems. This is because the amount of light received per pixel is limited by the performance of silicon (Si), which has insufficient light absorption characteristics owing to its indirect optical bandgap. Therefore, breakthrough technologies that can be used to drastically improve the sensitivity of image sensors are highly desirable.

The method of signal amplification through avalanche multiplication initiated by the impact ionization of photogenerated carriers is one of the most promising approaches to achieving high sensitivity. Avalanche multiplication in an amorphous selenium (a-Se) photoconversion layer was first utilized for image sensing in a high-gain avalanche rushing amorphous photoconductor (HARP) tube camera^4^. Because the excess noise of the avalanche decreases as the ratio of electron and hole ionization rates (*α*_*e*_ and *β*_*h*_) increases^5^, Se is advantageous over conventional Si in terms of its larger ratio of *β*_*h*_ to *α*_*e*_^6,7^. Unlike the original insulating structures with a-Se confined between two insulating layers that prevent the flow of both injected and photogenerated carriers^8^, the HARP incorporated new blocking structures consisting of wide-bandgap semiconductors. The new blocking structures prevent charge injection while providing a smooth flow of photogenerated carriers. Owing to the high avalanche gain and effective blocking structures, HARP cameras are characterized by ultrahigh sensitivity and low noise. Utilizing these features, they have been used for various applications such as high-sensitivity TV camera tubes^9^, low-dose X-ray imaging detectors^10–12^, and biomedical scientific cameras^13^. Researchers have attempted to incorporate a-Se-based HARP technologies into solid-state imagers^14–16^; however, there are still some challenges, including the high operation voltage required for avalanche multiplication and the vulnerability of a-Se to temperature changes^17,18^.

Crystalline-selenium (c-Se)-based photodiodes, which have an extremely high spectral response covering almost the entire visible region, are attractive alternatives to a-Se. Owing to the high optical absorption coefficient throughout the visible region^19^, the thickness of c-Se can be reduced to less than 500 nm, which is advantageous for low-voltage avalanche multiplication. In addition, among the several Se crystalline allotropes^20^, the hexagonal form, which we use in this work, is the most thermodynamically stable structure, which can ensure the reliability of devices^21^. Because the Se layers fabricated in this work are polycrystalline, there is an advantage and a disadvantage to applying them for imaging devices. The advantage is that polycrystalline Se can be fabricated on almost all types of substrates and the disadvantage is that crystal grains must be controlled to flatten the surface of the c-Se layer to obtain high-quality images. Previously, employing a wide-bandgap n-type semiconducting gallium oxide (Ga_2_O_3_) as a hole blocking layer, we reported avalanche multiplication at a low reverse bias voltage in a c-Se-based photodiode fabricated on a glass substrate, which exhibited extremely high external quantum efficiency (EQE) of more than 100%^22^.

In contrast to conventional CMOS image sensors, hybrid image sensors comprising non-Si photoconversion layers and CMOS field-effect transistors (CMOSFETs) have been reported to surpass the performance of Si photodiodes. By utilizing various characteristics of non-Si photoconversion films, hybrid image sensors have demonstrated many interesting performance features, including a high dynamic range^23^, a global-shutter mode^24^, and a high near-infrared (NIR)^25–30^ and X-ray sensitivity^31–34^. By taking advantage of the recent rapid progress in CMOS-stacking technologies, we previously fabricated c-Se-based hybrid CMOS image sensors to demonstrate high-spatial-resolution characteristics^35^. However, owing to an incomplete blocking component with the lack of an appropriate electron blocking layer, the photoconversion layer suffered from electron-injection-related dark current in the multiplication region^36^, which deteriorated the quality of a captured image. In addition, the captured image was filled with white spots caused by the local concentration of electric field at the edges of pixel electrodes.

In this study, we demonstrated c-Se-based hybrid 8 K resolution CMOS image sensors with a p-type wide bandgap nickel oxide (NiO) as a buffer and an electron blocking layer. Owing to the effective electric field relaxation and electron blocking effects of the defect-enhanced NiO, the photogenerated signals were amplified by a factor of approximately 1.4 while a low dark current of 2.6 nA/cm^2^ was maintained. On the basis of these developments, we successfully obtained a brighter image by avalanche multiplication without notable deterioration due to dark noise.

## Results and discussion

### Device structure with photoconversion layer

**Figure 1:**
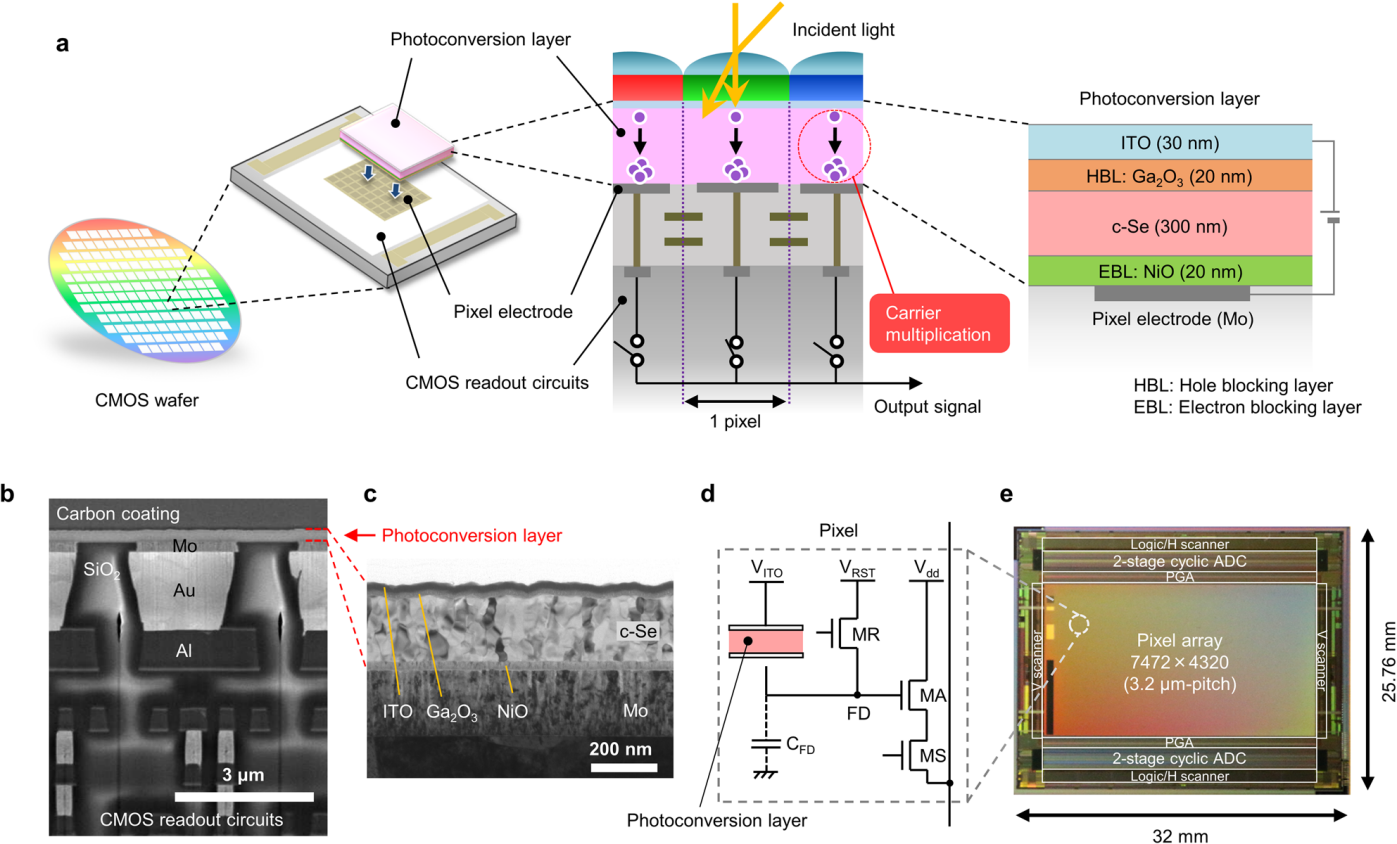
Device structure and pixel configuration. (**a**) Schematic cross section of the stacked CMOS image sensor with detailed photoconversion layer information. (**b**) Cross-sectional scanning electron microscopy image of a pixel. (**c**) Cross-sectional transmission electron microscopy image of a photoconversion layer. (**d**) Schematic diagram of a pixel. (**e**) Block diagram and photomicrograph of the chip.

A schematic cross section of the stacked CMOS image sensor with detailed information of the photoconversion layer is provided in Fig. 1a. The photoconversion layer, which consists of a c-Se light absorbing and multiplication layer, a Ga_2_O_3_ hole blocking layer, a NiO electron blocking layer, and an indium tin oxide (ITO) transparent electrode, was laminated on CMOS readout circuits connected through each pixel electrode. A cross-sectional scanning electron microscopy (SEM) image of a fabricated pixel and a cross-sectional transmission electron microscopy (TEM) image of a photoconversion layer are shown in Fig. 1b and c, respectively. First, a 20-nm-thick p-type wide-bandgap (*E*_*g*_: 4.0 eV^37^) NiO electron blocking layer was deposited directly on molybdenum (Mo) pixel electrodes by radio-frequency (RF) magnetron sputtering. Next, a tellurium (Te) nucleation layer and a 300-nm-thick a-Se layer were continuously deposited by thermal evaporation, followed by annealing to convert a-Se into a p-type c-Se^38^ (*E*_*g*_: 1.8 eV^39^). Then, a 20-nm-thick n-type wide-bandgap (*E*_*g*_: 4.8 eV^39^) Ga_2_O_3_ hole blocking layer was deposited by RF magnetron sputtering to form an n-Ga_2_O_3_/p–c-Se heterojunction. Finally, a 30-nm-thick ITO transparent electrode was deposited by direct current (DC) magnetron sputtering. Because designing the bandgap alignment is important for efficiently extracting the photogenerated carriers from the photoconversion layer to the external electrodes, we doped 8 at% tin (Sn) into Ga_2_O_3_ to reduce the conduction band offset (*ΔE*_*c*_) between Ga_2_O_3_ and c-Se from 0.8 eV (nondoped) to 0.5 eV (Supplementary Fig. 1a). The amount of Sn doped into Ga_2_O_3_ was analyzed by Rutherford backscattering spectrometry (RBS) and found to show a linear relationship with the tin oxide (SnO_2_) mole ratio in the Ga_2_O_3_-SnO_2_ sputtering target (Supplementary Fig. 1b). Herein the *E*_*g*_ of Ga_2_O_3_ decreases as the Sn doping concentration increases while keeping the valence band energy (*E*_*v*_) constant, as analyzed by X-ray photoelectron spectroscopy (XPS). As shown in Supplementary Fig. 1c, 20-nm-thick 8 at%-Sn-doped Ga_2_O_3_ still maintains high transparency of almost 90% in the visible region (400–700 nm), allowing most of the incident light to reach the c-Se absorbing layer. A potential energy diagram of the photoconversion layer is illustrated in Supplementary Fig. 2.

### Pixel configuration and signal readout

Figure 1d shows a schematic of a pixel of the fabricated image sensor. We fabricated a photoconversion layer on CMOS circuits, which we have previously reported^40^. The pixel has the typical three-transistor configuration consisting of a reset transistor (MR), an amplifying transistor (MA), and a select transistor (MS). In contrast to a conventional Si-based image sensor, the photoconversion element is replaced by a photoconversion layer that is electrically connected to CMOS readout circuits through a metal electrode. The photogenerated carriers in the photoconversion layer are transferred to the floating diffusion (FD) node and stored for a certain period. Then, the potential variation of the FD is amplified by a source follower and sequentially connected to the column output line through the MS. The block diagram and photomicrograph of the chip are shown in Fig. 1e. A schematic of the pixel electrode patterning process and an atomic force microscopy (AFM) image of the surface of the pixel arrays are respectively shown in Supplementary Fig. 3a and b. All pixel electrodes were embedded in the insulating silicon dioxide (SiO_2_) layer and the surface was flattened by chemical–mechanical polishing (CMP). The chip specifications are summarized in Supplementary Table 1. The developed image sensor has 8 K resolution (7472(H) × 4320(V) pixels) in a Super 35 mm optical format with a pixel size of 3.2 μm × 3.2 μm, a programmable gain amplifier (PGA), a two-stage cyclic analog-to-digital converter (ADC)^41,42^ with 12-bit resolution, a horizontal and vertical scanner, and a logic/output block.

### Buffering effects of NiO on decreasing the electric field concentration

NiO, which we newly applied here, has two crucial roles in resolving the problems encountered when developing high-performance photoconversion layers. We carried out a detailed investigation of these roles of NiO. Despite the many possible n-type wide-bandgap materials for hole blocking layers, there are few materials that meet the requirements for electron blocking layers, which need a p-type wide bandgap. NiO is a highly promising p-type transparent semiconductor. Owing to the specific characteristics of a wide optical bandgap and a wide-range of tunable carrier concentrations, NiO has been used in a variety of applications, such as visible–transparent UV photodetectors^43,44^, visible–transparent solar cells^45,46^, and UV–Vis light-emitting diodes^37,47,48^.

**Figure 2:**
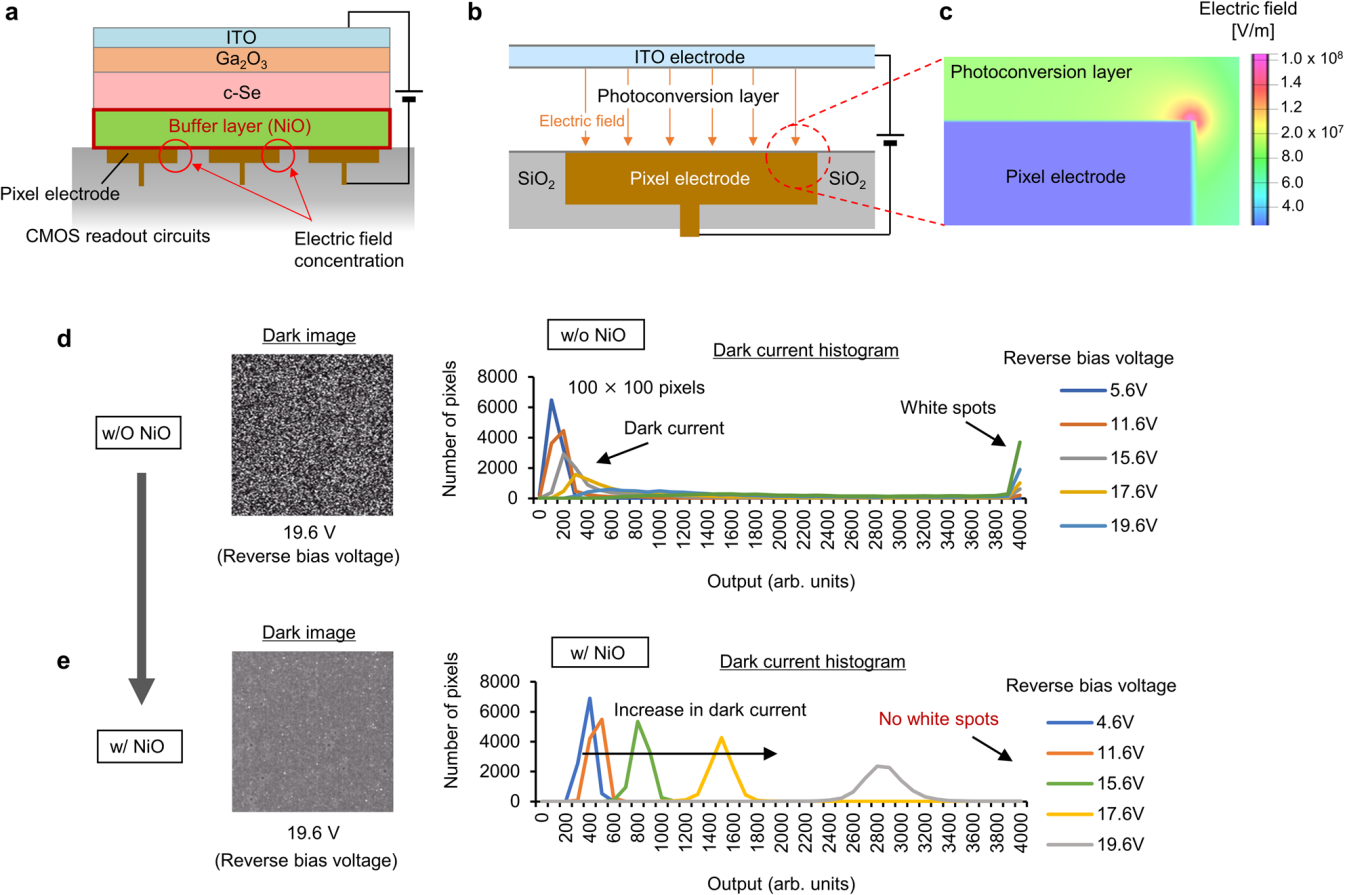
Electric field relaxation effects of NiO buffer layer. (**a**) Schematic of the electric field relaxation effects of NiO buffer layer. (**b**) Schematic cross-sectional illustration of the pixel model and **c** mapping of the calculated electric field gradient around a pixel electrode at an applied voltage of 3 V. Dark images (100 × 100 pixels) and their histograms with different reverse bias voltages captured by the stacked CMOS image sensors overlaid with photoconversion layers (**d**) without and (**e**) with NiO buffer layers, respectively.

Firstly, the insertion of NiO as a buffer layer directly into the CMOS readout circuits can reduce the concentration of electric field generated at the edge of each pixel electrode as shown in Fig. 2a. A schematic cross-sectional illustration of the pixel model and its mapping of the calculated electric field gradient around a pixel electrode at an applied voltage of 3 V are shown in Fig. 2b and c, respectively. The calculation results revealed that despite the ideal surface flatness of the pixels, the electric field at the edge of the electrode is approximately three times as strong as that at the surface. This extremely strong electric field induces partial electrical breakdown in the c-Se layer, leading to the emergence of white spots in the captured image. However, because the strength of the electric field decreases away from the edge, the introduction of a semi-insulating NiO layer effectively separates c-Se from the pixel electrodes to decrease the local concentration of the electric field. Figure 2d and e show a comparison of the dark images (100 × 100 pixels) extracted from the images captured by the fabricated image sensors overlaid with photoconversion layers with and without NiO buffer layers, respectively. A comparison of the dark current histograms obtained from the images, which correspond to the outputs of 12-bit (4096 greyscale level) images with different reverse bias voltages, is also shown in the figures. Without NiO, owing to the local concentration of the electric field adjacent to the edges of the pixel electrodes in c-Se, the dark image is filled with white spots with a reverse bias voltage of 19.6 V (Fig. 2d). Furthermore, the histogram also indicates the existence of white spots as pixels with saturated signals. On the other hand, in the case with NiO, the number of white spots was clearly decreased by separating c-Se from the edges of the pixel electrodes as shown in the dark image and its histogram (Fig. 2e). However, the histogram was shifted toward a higher output level which indicates an increase in dark current as the reverse bias voltage increases. Previously, it has been reported that the defect levels in blocking materials could lower the effective potential barrier^49,50^. Considering these reports, it is assumed that the electron injection-related dark current of a photoconversion layer increases owing to the insufficient potential barrier caused by the defect levels in NiO. In our initial experiments, because we deposited NiO by sputtering using NiO targets whose composition ratio of nickel (Ni) to oxygen (O) was 1:1, the introduction of excessive O_2_ increases the Ni vacancies and the deviation from the stoichiometry in NiO increased the defect levels, leading to a reduced electron blocking capability. Therefore, a precise control of the O_2_ concentration in NiO is required to suppress the electron injection.

### Electron blocking effects of NiO on decreasing the dark current

**Figure 3:**
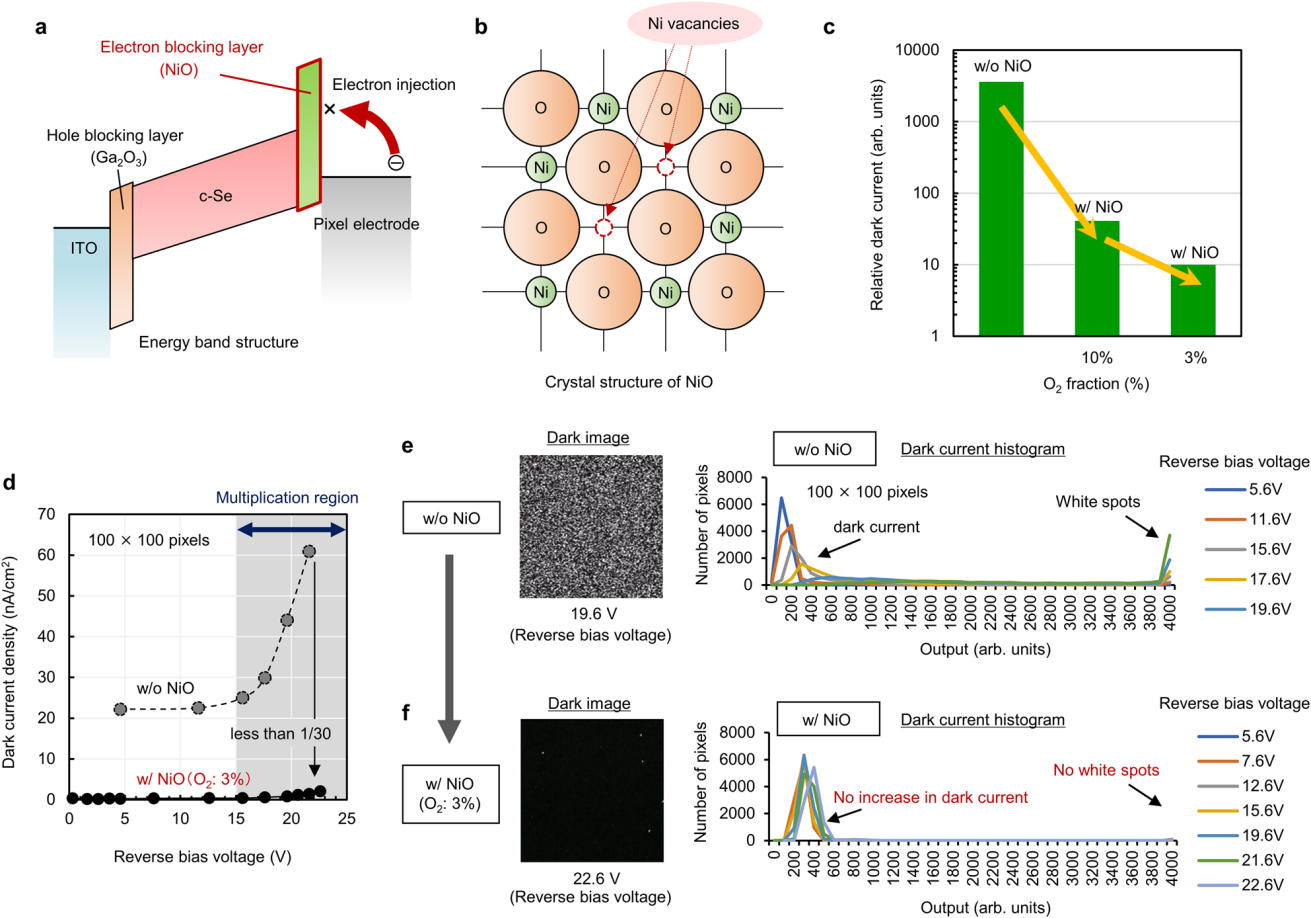
Effects of NiO electron blocking layer on dark current reduction. (**a**) Schematic of the effects of a NiO electron blocking layer on preventing electron injection from an external electrode. (**b**) Schematic diagram of the crystal structure of NiO with Ni vacancies. (**c**) Comparison of relative dark current of the test devices on glass substrates consisting of photoconversion layers without NiO and with NiO (O_2_ fractions of 10% and 3%). (**d**) Dark current density of the photoconversion layers with and without NiO (O_2_ fraction of 3%) on the CMOS readout circuits as a function of reverse bias voltage measured at room temperature. Dark images (100 × 100 pixels) and their histograms with different reverse bias voltages captured by the stacked CMOS image sensors overlaid with photoconversion layers (**e**) without and (**f**) with NiO (O_2_ fraction of 3%) electron blocking layers, respectively.

Secondly, the introduction of wide-bandgap NiO as a blocking layer between c-Se and pixel electrodes could effectively prevent electrons from being injected into the photoconversion layer as shown in Fig. 3a. Basically, NiO has a NaCl-type structure with the Ni^2+^ and O^2−^ ions in a 1:1 ratio interacting owing to a Coulomb force. However, NiO is likely to deviate from stoichiometry owing to the existence of excessive O_2_-induced Ni vacancies as schematically shown in Fig. 3b. For electrical neutralization, the number of Ni^3+^ replacing Ni^2+^ increases, which makes NiO exhibit p-type conductivity^51,52^. Therefore, to reduce the number of Ni vacancies, it is necessary to control the O_2_ concentration in NiO. Here, we deposited NiO by sputtering using a Ni metal target with a high purity of 99.999% (5 N) to control the O_2_ fraction more precisely. It was previously reported that the carrier concentration in NiO decreases as the O_2_ to argon (Ar) flow rate ratio in the sputtering gas decreases^53^. To investigate the effects of the O_2_ concentration in NiO on the dark current of the photoconversion layers, we fabricated test devices sandwiched between ITO electrodes on glass substrates as schematically illustrated in Supplementary Fig. 4. Figure 3c shows a comparison of the relative dark current of the test devices consisting of the photoconversion layers without and with NiO (O_2_ fraction in the sputtering gas of 10% and 3%). The results show that applying NiO as an electron blocking layer drastically decreases the dark current by several orders and the dark current becomes much lower with a smaller O_2_ fraction. Based on our previous research, though NiO becomes stoichiometric with a smaller O2 fraction of approximately 1%, as shown in the AFM 3D topographic images, NiO with an O_2_ fraction of 1% (Supplementary Fig. 5a) shows an approximately four times rougher surface morphology than NiO with an O_2_ fraction of 3% (Supplementary Fig. 5b). The deposition rate of NiO increases with a decrease in the O_2_ fraction during the deposition of NiO at the same sputtering power. Owing to the larger grain size with a faster deposition rate, the surface of NiO becomes rougher with a smaller fraction of O_2_. Because the Se layer grows nonuniformly while crystallizing on the roughened NiO surface, we decided to apply an O_2_ fraction of 3% during NiO deposition. Then, we applied this deposition condition to the fabrication of stacked image sensors overlaid with a photoconversion layer. Figure 3d shows a comparison of the dark current density of the photoconversion layer with and without^36^ NiO on the CMOS readout circuits as a function of reverse bias voltage measured at room temperature. The dark current was estimated from partial dark images of 100 × 100 pixels extracted from 8 K captured images. As can be seen in the figure, the dark current sharply decreased from 60 nA/cm^2^ to 1.5 nA/cm^2^ at a reverse bias voltage of 21.6 V in the multiplication region upon applying NiO with an O_2_ fraction of 3%. The dark images (100 × 100 pixels) without and with optimized NiO at reverse bias voltages of 19.6 V and 22.6 V are shown in Fig. 3e and f, respectively, and histograms with different reverse bias voltages are also shown. By applying the low-defect-density NiO provided through the accurate control of the O_2_ supply, in addition to preventing the appearance of white spots owing to the buffering effects, the increase in dark current was successfully suppressed owing to the strong electron blocking effect.

### Enhancement of image sensing by avalanche multiplication

With the application of the improved NiO as a buffer and an electron blocking layer, we further studied the photoconversion characteristics of the photoconversion layer stacked onto the CMOS readout circuit. For the device fabrication, in consideration of the effects of the surface morphologies of the c-Se layers on the quality of the captured images as mentioned above, we simply prepared c-Se on the Si substrates crystallized at different temperatures, whose AFM images are shown in Supplementary Fig. 6a. The mean roughness (Ra) and maximum roughness (Rmax) as a function of Se crystallization temperature are shown in Supplementary Fig. 6b. As can be seen from the figure, both Ra and Rmax decrease as the crystallization temperature increases up to 160 °C, above which they increase up to 200 °C. Therefore, we selected c-Se-based photoconversion layers crystallized at 160 °C and 200 °C on the CMOS readout circuits to compare the image quality. The images captured with the c-Se-based photoconversion layers crystallized at 200 °C (Supplementary Fig. 7a) and 160 °C (Supplementary Fig. 7b) and their histograms (100 × 100 pixels) are shown in the figures. The dispersion of the histogram in Supplementary Fig. 7b is considerably smaller than that of the histogram in Supplementary Fig. 7a, which indicates that a flatter c-Se surface is needed to obtain a high-quality image. Because the crystallization temperature has little effect on crystallinity, we optimized the crystallization temperature depending on the surface roughness.

**Figure 4:**
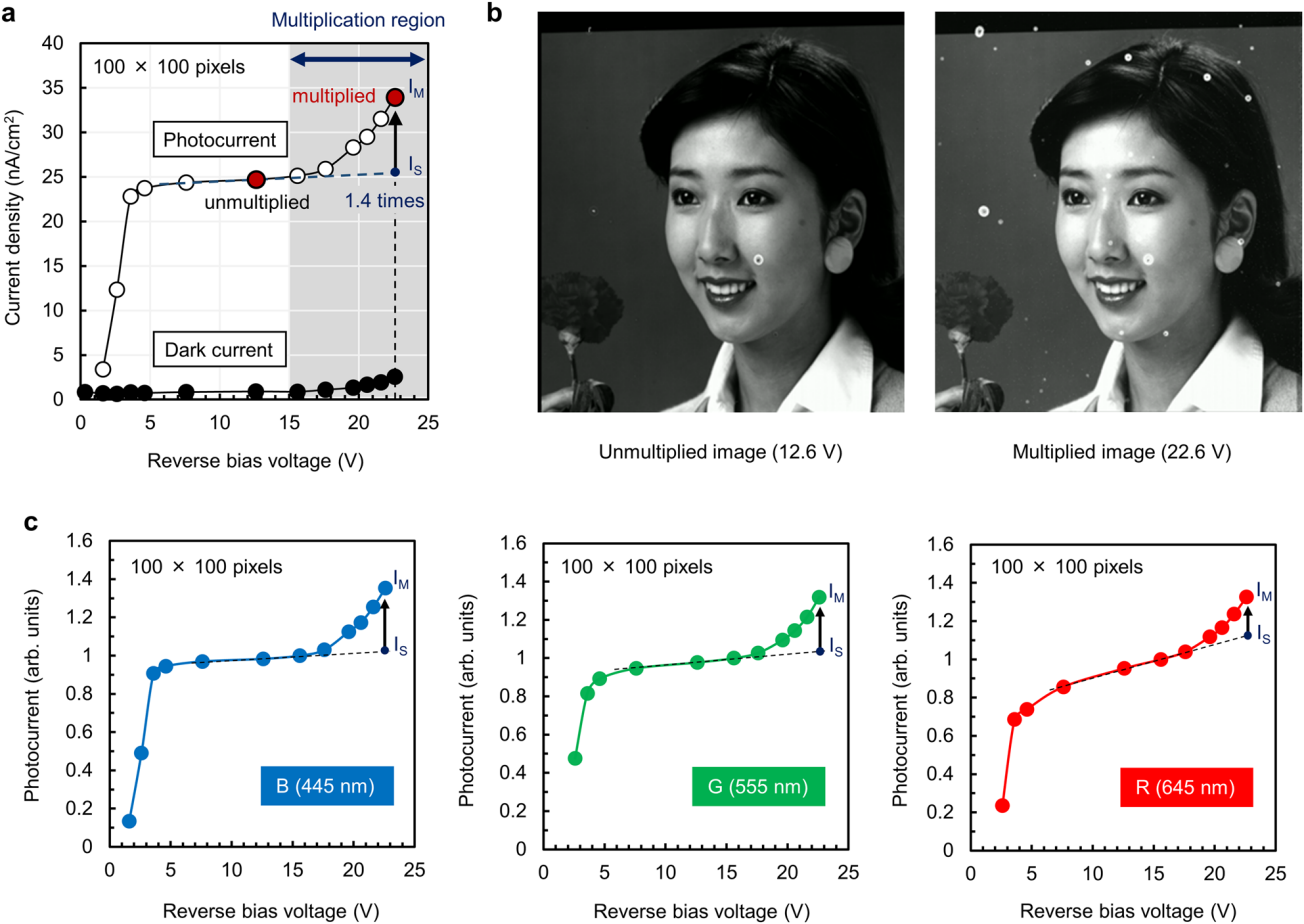
Electric field relaxation effects of NiO buffer layer. (**a**) Current density–voltage characteristics of a photoconversion layer with NiO (O_2_ fraction of 3%) stacked onto the CMOS readout circuit, in the dark and illuminated through a blue filter. (**b**) Partial images extracted from the original 8 K images captured by the stacked CMOS image sensors at reverse bias voltages of 12.6 V (unmultiplied) and 22.6 V (multiplied). (**c**) Normalized photocurrent–voltage characteristics of the stacked CMOS image sensors for the illuminated conditions through the blue, green, and red filters.

The measured current density–voltage characteristics of a photoconversion layer with NiO (O_2_ fraction of 3%) stacked onto the CMOS readout circuit, in the dark and illuminated through a blue filter, are presented in Fig. 4a. As shown in the figure, the photocurrent, which is calculated by subtracting the dark current from the measured signals, rapidly increases and becomes saturated above 5 V as the reverse bias voltage increases. When the reverse bias voltage is further increased to above 15 V, the photocurrent increases again with avalanche multiplication. Here, the multiplication factor *M* is defined as the ratio of *I*_*M*_ to *I*_*S*_ (*I*_*M*_/*I*_*S*_), where *I*_*M*_ is the practical multiplied photocurrent and *I*_*S*_ is the photocurrent obtained by the extrapolation of the approximate line for the signals in the saturation region at the same reverse bias voltage as indicated in the figure. At a reverse bias voltage of 22.6 V, *M* was calculated to be approximately 1.4 (*I*_*M*_: 34 nA/cm^2^, *I*_*S*_: 25 nA/cm^2^), while the dark current remained only 2.6 nA/cm^2^. Figure 4b shows partial images extracted from the original 8 K images captured by the fabricated image sensors at reverse bias voltages of 12.6 V (unmultiplied) and 22.6 V (multiplied). Owing to the effective electric field buffering and electron blocking effects of the optimized NiO, we successfully obtained a brighter image by avalanche multiplication without notable deterioration due to dark noise. The normalized photocurrent–voltage characteristics in the case of illumination through the blue, green, and red filters are shown in Fig. 4c. The normalized spectral transmittance of the color filters with central wavelengths of 445 nm (blue), 555 nm (green), and 645 nm (red) is shown in Supplementary Fig. 8a. Because the light penetration depth for each wavelength depends on the optical absorption coefficient *α* of c-Se (Supplementary Fig. 8b), the light intensity *I*(*x*) at the light propagation length *x* in a film is described by

1 $$ I\left( x \right) \, = I_{0} \times {\text{ exp}}\left( { - \alpha x} \right), $$

where *I*_*0*_ is the incident light intensity. From Supplementary Fig. 8b, *α* is revealed to be 3.2 × 10^5^ cm^−1^ for 445 nm, 1.8 × 10^5^ cm^−1^ for 555 nm, and 3.6 × 10^4^ cm^−1^ for 645 nm. Half the penetration depth (the distance at which the light intensity falls to 1/2e), for example, is calculated to be 15 nm for 445 nm, 28 nm for 555 nm, and 140 nm for 645 nm. Thus the effective hole multiplication length *L* in 300-nm-thick c-Se is determined to be 285 nm (*L*_*B*_), 272 nm (*L*_*G*_), and 160 nm (*L*_*R*_), respectively. Next, the multiplication factor *M* for holes is described by^54^

2 $$ M = {\text{ exp}}\left( {\beta_{h} L} \right), $$

where *β*_*h*_ is the hole-ionization coefficient. From Eq. (2), ln(*M*_*B*_)/ln(*M*_*G*_) = *L*_*B*_/*L*_*G*_ and ln(*M*_*B*_)/ln(*M*_*R*_) = *L*_*B*_/*L*_*R*_ are derived, where *M*_*B*_ is for blue, *M*_*G*_ is for green, and *M*_*R*_ is for red. As can be seen from Fig. 4c, *M*_*B*_, *M*_*G*_, and *M*_*R*_ are estimated to be 1.35 (*I*_*M*_/*I*_*S*_ = 1.36/1.01), 1.31 (*I*_*M*_/*I*_*S*_ = 1.32/1.01), and 1.19 (*I*_*M*_/*I*_*S*_ = 1.33/1.13), respectively. As a result, ln(*M*_*B*_)/ln(*M*_*G*_) (= 1.11) and ln(*M*_*B*_)/ln(*M*_*R*_) (= 1.81) are approximately in agreement with *L*_*B*_/*L*_*G*_ (= 1.05) and *L*_*B*_/*L*_*R*_ (= 1.78), respectively. These results indicate that the *M* values for each wavelength experimentally determined from the photocurrent–voltage characteristics are consistent with the estimation calculated from the effective hole multiplication length *L*, which confirms that avalanche multiplication is the dominant gain mechanism in the photoconversion layers.

## Conclusion

In this work, we have demonstrated avalanche-type stacked image sensors with a hybrid structure consisting of a c-Se-based photoconversion layer and CMOSFETs by addressing the problems of the appearance of white spots and the increase in dark current under a high electric field. Introducing NiO directly into the CMOS readout circuits as a buffer and an electron blocking layer resolved these crucial problems. The bandgap alignment (Supplementary Fig. 2) of the proposed layer configuration including wide-bandgap NiO is ideal for the blocking structures; however, the high electron barrier capability is not achieved until Ni vacancy-induced defect levels are eliminated. By controlling the O_2_ fraction during NiO sputtering to decrease the defect levels, the dark current in the fabricated image sensor with NiO (O_2_ fraction of 3%) decreased to less than 1/30 that without NiO at a reverse bias voltage of 21.6 V. Subsequently, we confirmed signal amplification by a multiplication factor *M* of approximately 1.4 while the dark current remained as low as 2.6 nA/cm^2^ at a reverse bias voltage of 22.6 V. Moreover, we successfully reproduced a brighter image based on the amplified signals without any notable noise degradation. The wavelength dependence of multiplication gains experimentally determined from photocurrent–voltage measurements showed good agreement with values estimated from the relational equations for effective hole multiplication length, confirming that avalanche multiplication is the dominant gain mechanism in the photoconversion layers. In addition, all the layer fabrication processes were implemented at a relatively low temperature (< 200 °C) and are compatible with conventional CMOS manufacturing processes. Our approaches to improving the sensitivity of image sensors are expected to particularly contribute to high-end image sensing applications with a large number of fine pixels. We believe that further investigation of the details of avalanche multiplication phenomena will open up new perspectives for highly sensitive image sensing.

## Methods

### Device fabrication

CMOS readout circuits with patterned Mo pixel electrodes (Supplementary Fig. 3) were used for device fabrication. After dicing the wafer into chips, a 20-nm-thick electron blocking NiO layer was deposited directly onto the CMOS readout circuits by RF sputtering using pure Ni targets (Ni, 99.999%) at 0.1 Å/s with a sputtering power of 100 W. After depositing NiO, a thin Te nucleation layer was deposited by thermal evaporation using 99.999% purity Te pellets at 0.02 Å/s to prevent the Se layer from peeling during annealing. Then a 300-nm-thick a-Se layer was deposited by thermal evaporation using 99.999% purity Se pellets at 8.0 Å/s, followed by annealing at 16 °C for 1 min in air to convert a-Se into c-Se. Next, a 20-nm-thick hole blocking 8 at%-Sn-doped Ga_2_O_3_ layer was deposited by RF sputtering using targets (99.99%) composed of 60 mol% Ga_2_O_3_ and 40 mol% SnO_2_ at 0.7 Å/s with a sputtering power of 100 W. After annealing at 160 °C, a 30-nm-thick ITO layer was deposited to serve as a transparent electrode by DC sputtering using ITO targets (99.99%) composed of 95 mol% indium oxide (In_2_O_3_) and 5 mol% SnO_2_. The voltage was supplied to the photoconversion layer through the ITO contact pad, and the reverse bias was defined as the ITO electrode being positive with respect to the pixel electrode.

### Device characterization

The evaporation rates and the thicknesses of Te and c-Se were in situ monitored by a quartz crystal microbalance. The thicknesses of NiO, Ga_2_O_3_, and ITO were measured by X-ray reflectometry (XRR) performed using a Rigaku SmartLab diffractometer (CuKα, 45 kV, 200 mA) in the reflectometry mode. The ionization potential was measured by X-ray photoelectron spectroscopy (XPS) with spectra recorded using a PHI Quantera SXM XPS spectrometer with a monochromatic Al Kα radiation source. The optical bandgap (*E*_*g*_) was determined by extrapolating the (*αhυ*)^2^ versus *hν* Tauc plot. The amount of Sn doped into Ga_2_O_3_ was analyzed by Rutherford backscattering spectrometry (RBS) performed using 2 meV 4He^+^ induced RBS (CEA). A cross-sectional scanning electron microscopy (SEM) image of a pixel of the fabricated image sensor was obtained using an FEI Versa 3D DualBeam SEM operated at 5 kV. A cross-sectional transmission electron microscopy (TEM) image of a photoconversion layer was obtained using an FEI Tecnai F20 operated at 200 kV. Both SEM and TEM samples were prepared using an FEI Versa 3D focused ion beam (FIB). Surface morphology images of the c-Se films were obtained by a Bruker atomic force microscopy (AFM) system in the tapping mode. The dark current–voltage characteristics of the test devices on the glass substrates were analyzed using a Keithley 4200-SCS source measure unit. UV–Vis transmission spectra were measured using a multi-channel photo detector (Otsuka Electronics, MCPD–3700). The SIMULIA Opera FEA software (Version: Opera 16R1, https://www.3ds.com/products-services/simulia/products/opera/) developed by Dassault Systems was used to carry out the simulations on the electric field gradient around a pixel electrode.

### Informed consent

The images in Fig. 4b and Supplementary Fig. 7a and b were selected from the imaging standard charts distributed by the Institute of Image Information and Television Engineers (ITE) https://www.ite.or.jp/content/test-materials/. The images were permitted to publish under a CC BY open access license by the copyright holder (ITE).

## Supplementary Information

Supplementary Information

## Data Availability

The data that support the findings of this study are available from the corresponding author upon reasonable request.
