# Supplementary material for: Enhanced image sensing with avalanche multiplication in hybrid structure of crystalline selenium photoconversion layer and CMOSFETs
Source: Sci Rep. 2020 Dec 14;10:21888. doi: 10.1038/s41598-020-78837-7 (PMC7736349; doi:10.1038/s41598-020-78837-7)
Supplement: Supplementary file 1 — Supplementary Information [file 41598_2020_78837_MOESM1_ESM.pdf]

Supplementary Information

Enhanced image sensing with avalanche multiplication in hybrid structure of crystalline selenium photoconversion layer and CMOSFETs

Shigeyuki Imura[1*], Keitada Mineo[1], Yuki Honda[2], Toshiki Arai[1], Kazunori Miyakawa[1], Toshihisa Watabe[1], Misao Kubota[1], Keisuke Nishimoto[3], Mutsumi Sugiyama[3] & Masakazu Nanba[1]

[1]*Japan Broadcasting Corporation (NHK) Science & Technology Research Laboratories, 1-10-11 Kinuta, Setagaya-ku, Tokyo 157-8510, Japan*

[2]*Japan Broadcasting Corporation (NHK) Engineering System Inc., 1-10-11 Kinuta, Setagaya-ku, Tokyo 157-8510, Japan*

[3]*Department of Electrical Engineering, Faculty of Science and Technology, Tokyo University of Science, 2641 Yamazaki, Noda, Chiba 278-8510, Japan*

*email: imura.s-la@nhk.or.jp

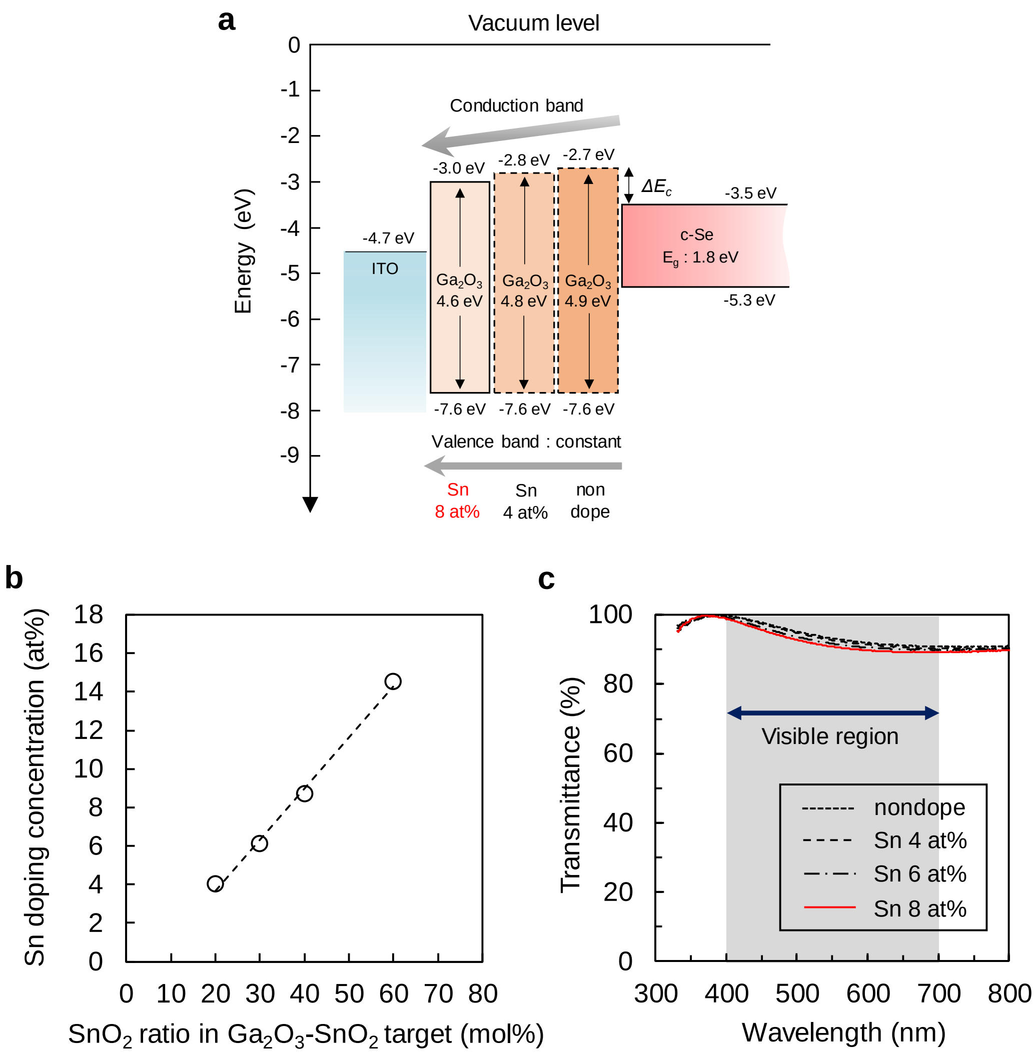

Supplementary Fig. 1. Analysis and characterization of Ga_2O_3 with different Sn doping concentrations a Energy band diagram of the photoconversion layer with Ga_2O_3 for different Sn doping concentrations. **b** Sn doping concentrations of Ga_2O_3 deposited on Si substrates analyzed by RBS as a function of SnO_2 ratio in Ga_2O_3-SnO_2 sputtering targets. **c** Transmission spectra of Ga_2O_3 with different Sn doping concentrations.

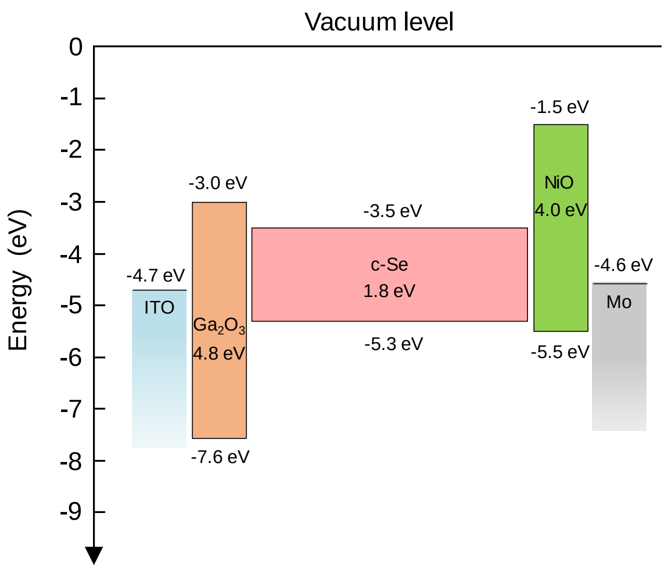

Supplementary Fig. 2. Energy band diagram of photoconversion layer with hole and electron blocking layers.

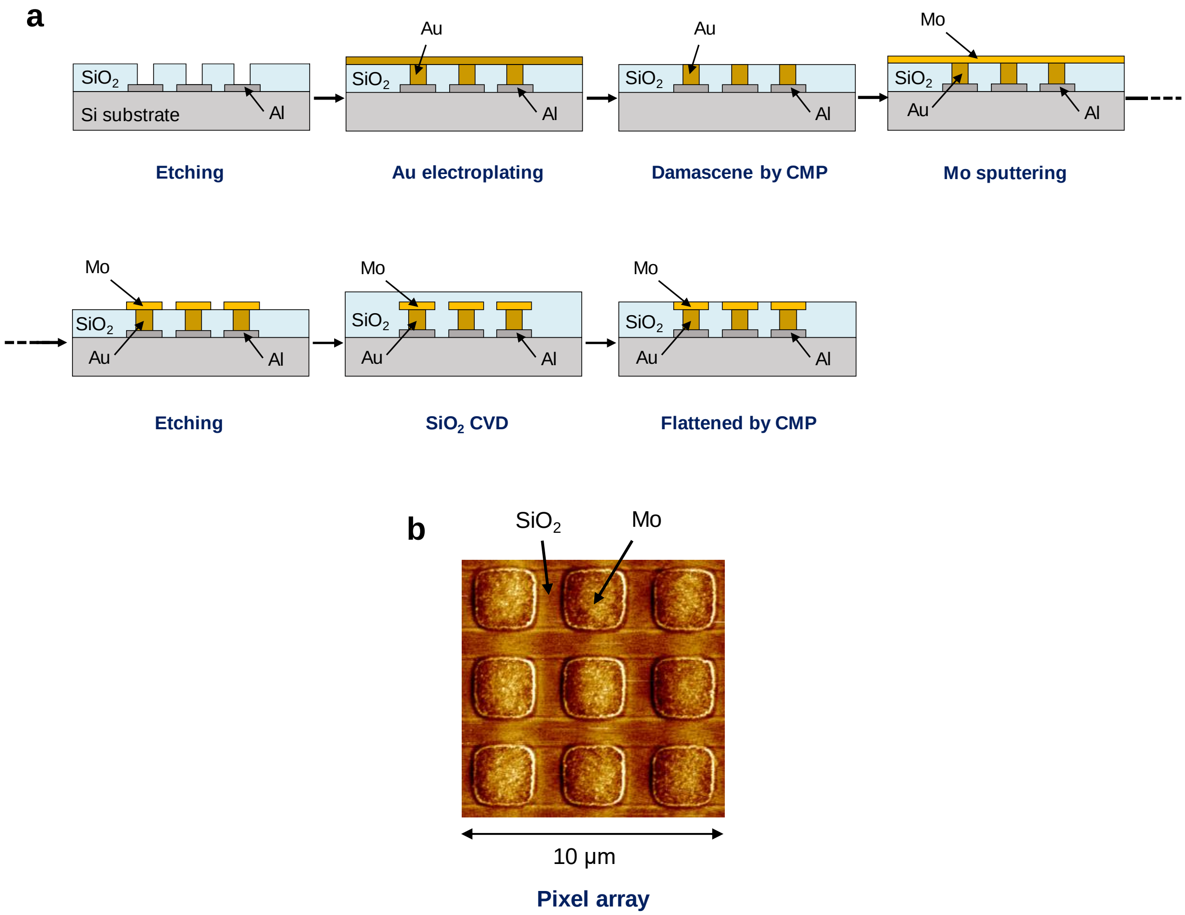

Supplementary Fig. 3. Pixel electrodes of the CMOS readout circuits. a Schematic of the pixel electrode patterning process. **b** Atomic force microscopy image of the surface of the pixel arrays.

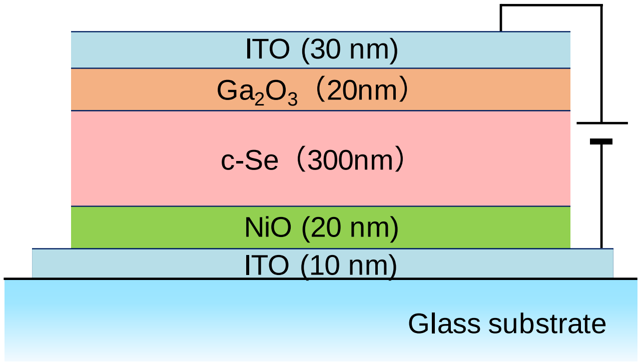

Supplementary Fig. 4. Schematic cross section of a photoconversion layer of a test device on a glass substrate.

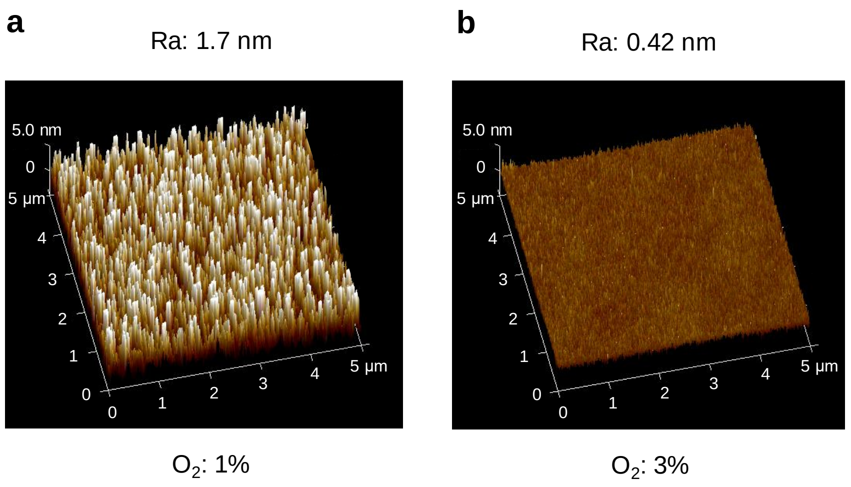

Supplementary Fig. 5. Surface morphology of NiO with different O_2 fractions. Atomic force microscopy images and mean roughness (Ra) of the surface of NiO with O_2 fractions of **a** 1% and **b** 3%.

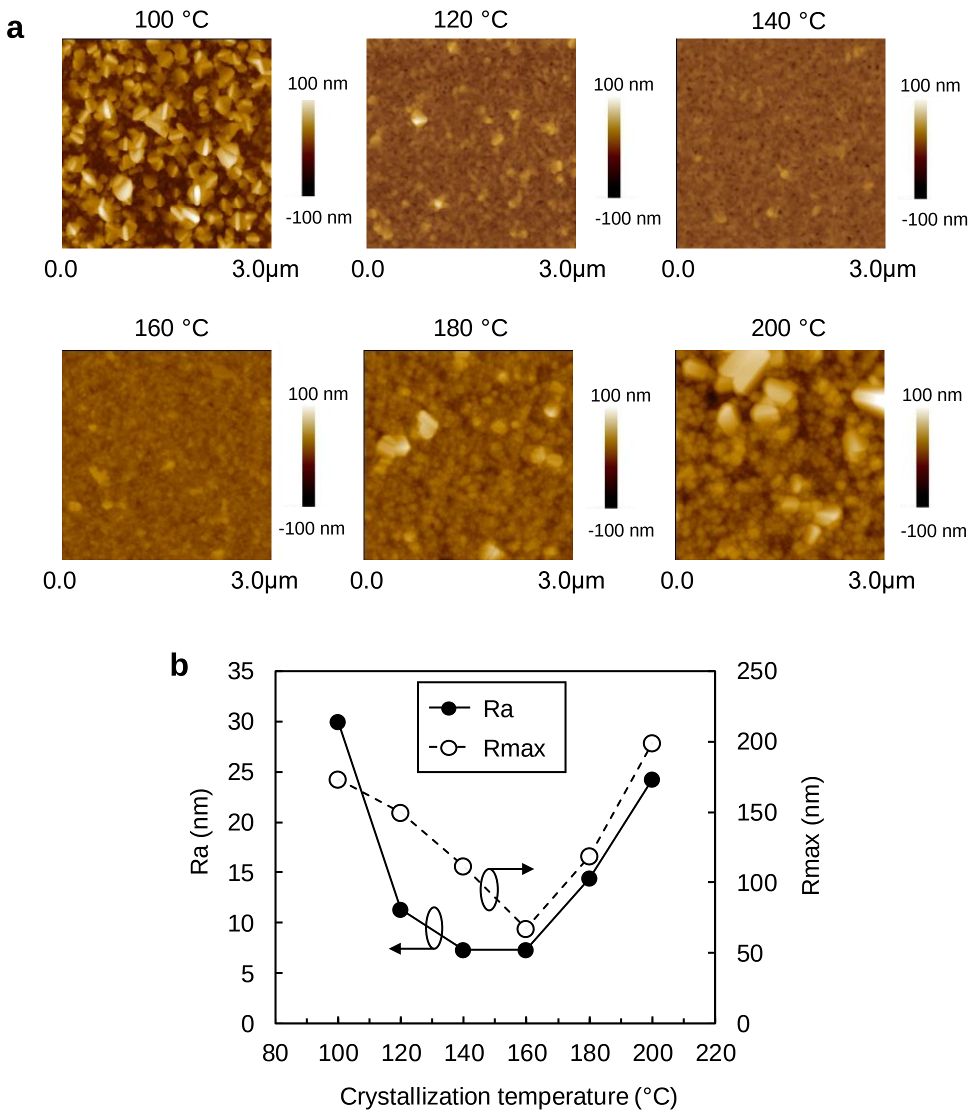

Supplementary Fig. 6. Surface morphology of c-Se with different crystallization temperatures. a Atomic force microscopy images of the surface of c-Se fabricated at different crystallization temperatures. **b** Mean roughness (Ra) and maximum roughness (Rmax) as a function of crystallization temperature.

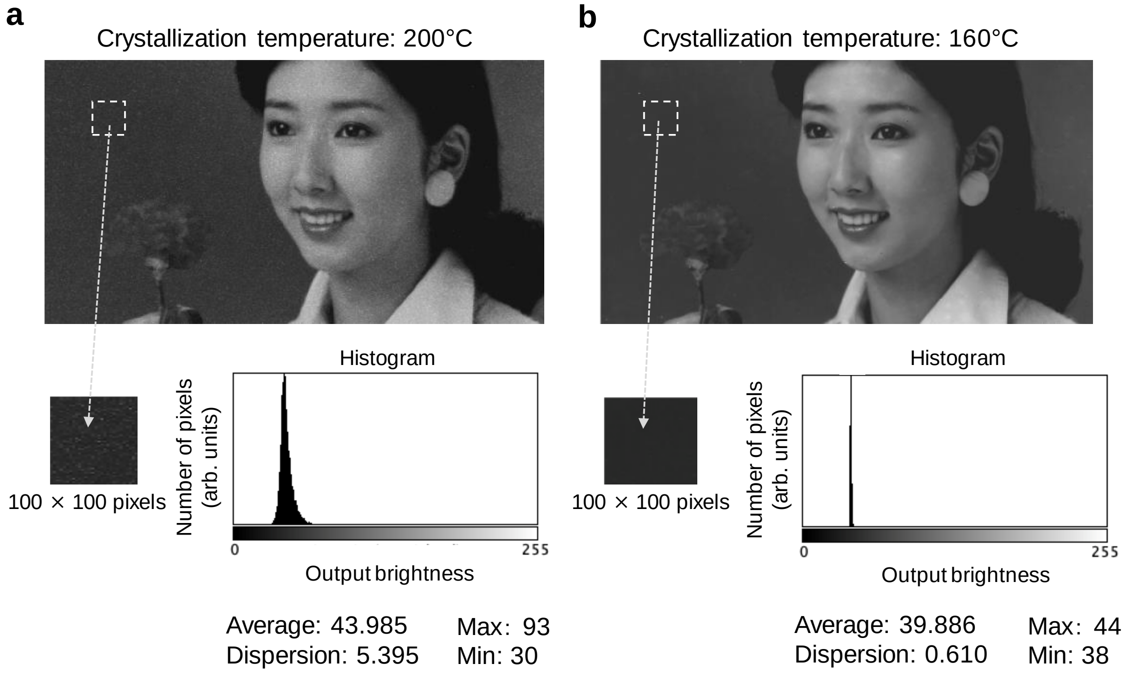

Supplementary Fig. 7. Evaluation of image quality obtained from stacked image sensors fabricated with different Se crystallization temperatures. Captured Images (100 × 100 pixels) and their histograms with c-Se photoconversion layers crystallized at **a** 200°C and **b** 160°C.

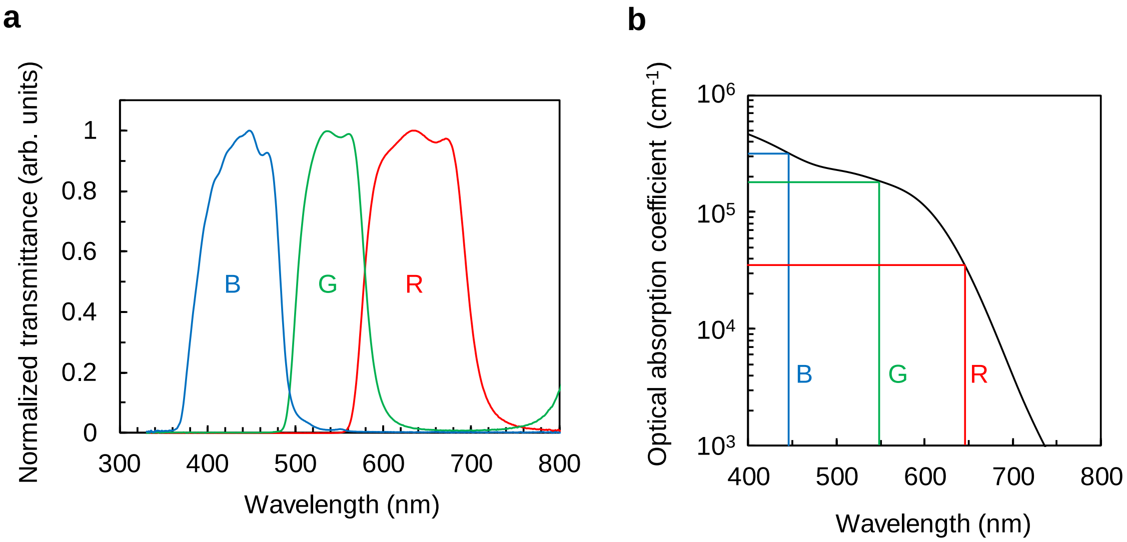

Supplementary Fig. 8. Absorption coefficient of c-Se with different wavelengths through color filters. a Normalized transmission spectra of blue, green, and red color filters. **b** Absorption spectra for c-Se.

Supplementary Table 1. Chip specifications.

Parameter	Value
Number of total pixels	7816(H) × 4360(V)
Number of effective pixels	7472(H) × 4320(V)
Pixel size	3.2 μm × 3.2 μm
Pixel type	3 transistors
ADC	2-stage cyclic
Resolution	12 bit
Frame frequency	60 Hz
Optical format	Super 35mm
Chip size	32 mm(H) × 25.76 mm(V)
Power consumption	3.0 W
